# Design and user experience testing of a polygenic score report: a qualitative study of prospective users

**DOI:** 10.1186/s12920-021-01056-0

**Published:** 2021-10-01

**Authors:** Deanna G. Brockman, Lia Petronio, Jacqueline S. Dron, Bum Chul Kwon, Trish Vosburg, Lisa Nip, Andrew Tang, Mary O’Reilly, Niall Lennon, Bang Wong, Kenney Ng, Katherine H. Huang, Akl C. Fahed, Amit V. Khera

**Affiliations:** 1 Center for Genomic Medicine, Department of Medicine, Massachusetts General Hospital, 185 Cambridge Street, Simches Research Building | CPZN 6.256, Boston, MA 02114 USA; 2 Program in Medical and Population Genetics, Broad Institute of MIT and Harvard, Cambridge, MA USA; 3 Pattern Visualization Team, Broad Institute of MIT and Harvard, Cambridge, MA USA; 4 Center for Computational Health, IBM Research, Cambridge, MA USA; 5 Genomics Platform, Broad Institute of MIT and Harvard, Cambridge, MA USA; 6 Department of Medicine, Harvard Medical School, Boston, MA USA

**Keywords:** Polygenic scores, Laboratory reports, Patient communication, Population health, Data visualization, Health communication, Genomic medicine

## Abstract

**Background:**

Polygenic scores—which quantify inherited risk by integrating information from many common sites of DNA variation—may enable a tailored approach to clinical medicine. However, alongside considerable enthusiasm, we and others have highlighted a lack of standardized approaches for score disclosure. Here, we review the landscape of polygenic score reporting and describe a generalizable approach for development of a polygenic score disclosure tool for coronary artery disease.

**Methods:**

We assembled a working group of clinicians, geneticists, data visualization specialists, and software developers. The group reviewed existing polygenic score reports and then designed a two-page mock report for coronary artery disease. We then conducted a qualitative user-experience study with this report using an interview guide focused on comprehension, experience, and attitudes. Interviews were transcribed and analyzed for themes identification to inform report revision.

**Results:**

Review of nine existing polygenic score reports from commercial and academic groups demonstrated significant heterogeneity, reinforcing the need for additional efforts to study and standardize score disclosure. Using a newly developed mock score report, we conducted interviews with ten adult individuals (50% females, 70% without prior genetic testing experience, age range 20–70 years) recruited via an online platform. We identified three themes from interviews: (1) visual elements, such as color and simple graphics, enable participants to interpret, relate to, and contextualize their polygenic score, (2) word-based descriptions of risk and polygenic scores presented as percentiles were the best recognized and understood, (3) participants had varying levels of interest in understanding complex genomic information and therefore would benefit from additional resources that can adapt to their individual needs in real time. In response to user feedback, colors used for communicating risk were modified to minimize unintended color associations and odds ratios were removed. All 10 participants expressed interest in receiving a polygenic score report based on their personal genomic information.

**Conclusions:**

Our findings describe a generalizable approach to develop a polygenic score report understandable by potential patients. Although additional studies are needed across a wider spectrum of patient populations, these results are likely to inform ongoing efforts related to polygenic score disclosure within clinical practice.

**Supplementary Information:**

The online version contains supplementary material available at 10.1186/s12920-021-01056-0.

## Background

The predictive capacity of polygenic scores—which quantify inherited risk by integrating information from many common sites of DNA variation—have improved considerably in recent years, accelerating consideration of adoption in clinical medicine [1]. Several commercial and academic groups have launched efforts to compute and report polygenic scores to patients or consumers. However, approaches for developing and reporting polygenic scores have been highly variable [2, 3]. While there have been efforts to standardize reporting of polygenic score development, best practice guidelines for clinical reporting do not yet exist.

The traditional approach to genetic testing and report development has focused on rare, monogenic variants [4–9]; polygenic score reporting has at least three important conceptual differences. First, polygenic score results are calculated based on hundreds to millions of DNA variants and reported as continuous rather than binary variables. Therefore, they cannot be summarized using Human Genome Variation Society nomenclature and classified by pathogenicity as is recommended for monogenic reports by current clinical guidelines [10–13]. Prior recommendations suggest use of a neutral statement of fact to summarize genetic results, such as ‘a change in gene XYZ was found’ [14]. However, since a polygenic score can only be calculated based on genome-wide variation and interpreted in the context of a population reference distribution, a parallel statement does not exist.

Second, consensus recommendations for clinical management based on polygenic scores are not yet available. For monogenic conditions such as a pathogenic *BRCA1* variant predisposing to breast and other cancers, expert consensus guidelines from groups such as the National Comprehensive Cancer Network and U.S. Preventive Services Task Forces are available [15, 16]. Additional efforts—ideally informed by prospective studies of clinical utility across ancestrally diverse populations—are needed to guide clinical decision support and management based on polygenic scores.

Third, most studies to date have described the impact of polygenic scores in relative terms (e.g., ‘XX-fold increased risk compared to population average’), but most clinical decision making is based on absolute risk assessment. The ‘penetrance’ of a high polygenic score—the probability of developing disease—is not fully known, and few tools that integrate polygenic scores with monogenic or non-genetic risk factors are currently available for clinical use.

We recognized an unmet need for research on design of polygenic score reports, risk disclosure tools, and accompanying educational resources that could be understood by both patients and non-expert clinicians. Here, we describe a generalizable approach for design of polygenic score reports using a user-centric approach, conduct a qualitative research study of user understanding and experience, and discuss new media that could enable a personalized and interactive experience with results from polygenic risk models.

## Methods

### Working group for polygenic score report for coronary artery disease

**Fig. 1:**
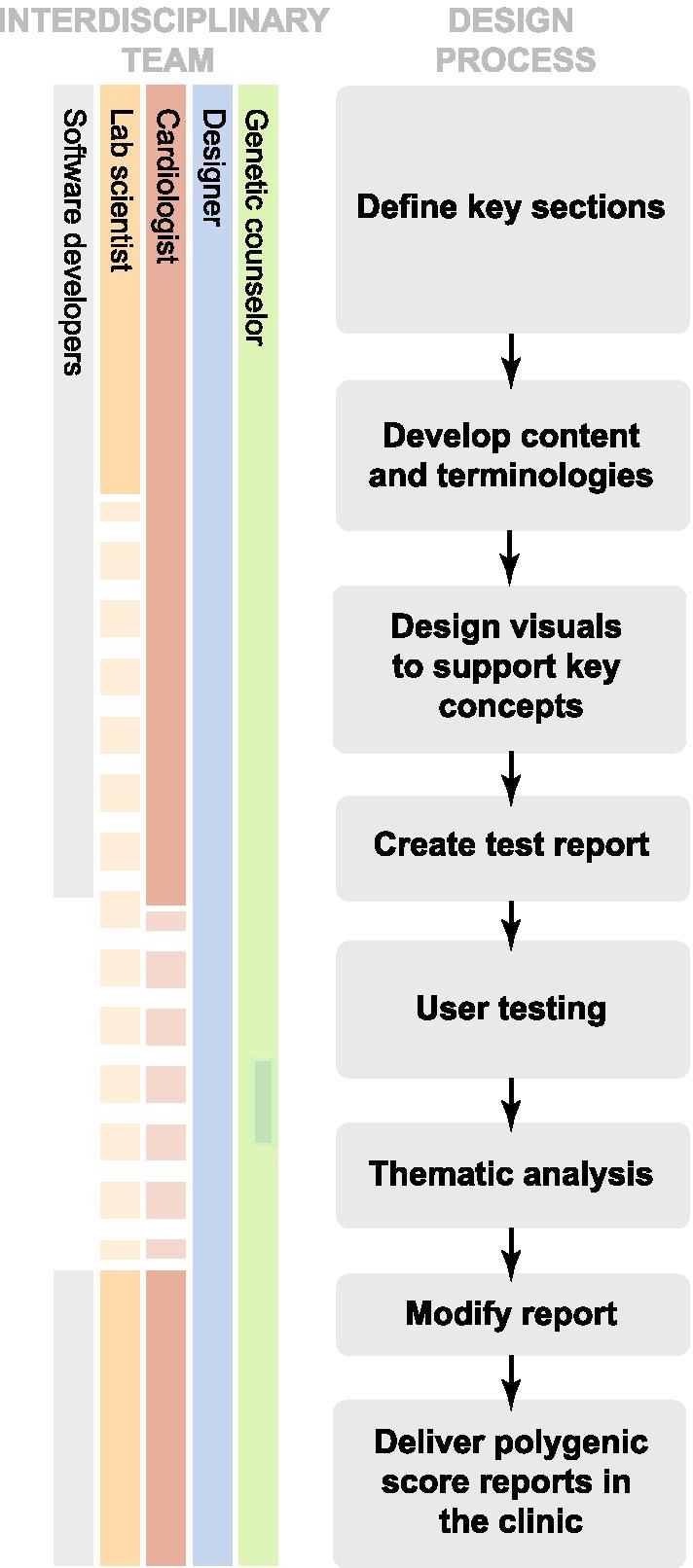
Developing a user-centered polygenic score report. An interdisciplinary team adopted a multi-step approach to create and iterate on a polygenic score report for coronary artery disease through a review of existing polygenic score reports and qualitative research methods

To develop a polygenic score report for coronary artery disease (CAD), we assembled a working group of 2 practicing physicians, 1 genetic counselor, 2 clinical genetic laboratory experts, 2 designers, and 1 software developer. We selected CAD as a representative example of an important and preventable complex disease where polygenic scores have been well-validated [17–19]. Over a period of 6 months, members of the group worked on iterative design and content creation of a draft polygenic score report for CAD (Fig. 1).

### Review of the current landscape of polygenic score reports

**Fig. 2:**
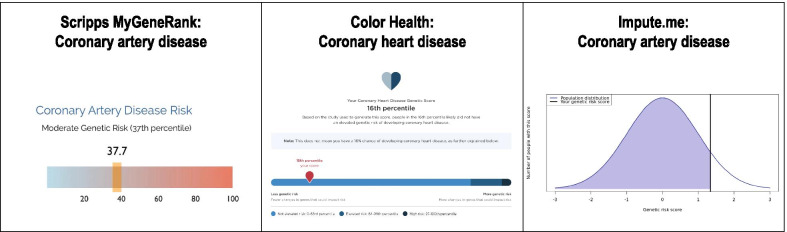
Comparison of polygenic risk score report visuals. Polygenic risk scores were compared based on numeric estimates reported, risk descriptions, and supporting visuals to convey risk. Written copyright permission was obtained from 3/7 groups to reproduce figures from company websites and provided through personal communication in this manuscript. References for sample polygenic score reports shown here: Scripps MyGeneRank [29], Color Health [32], Impute.me [34]. Copyright permissions were not obtained for the remaining report visuals discussed in the manuscript; sample reports are referenced within the manuscript: Myriad Genetics [27], Gene Plaza [33], 23andMe [36], Ambry Genetics [22–24]. Since this review, Ambry Genetics [22–24] removed the ‘AmbryScore’ polygenic score product from the market in May 2021 [e-mail communication]

Members of the working group (DGB, LP) reviewed nine publicly available polygenic score reports from seven groups; reports were identified through PubMed, internet search, the Polygenic Score Catalog [20], or provided through personal communication (Fig. 2). Since this review, one company removed their polygenic score product from the market because polygenic scores ‘have not been validated for use in patients of diverse backgrounds’ (e-mail communication with Ambry Genetics). Among these seven groups, CAD was the most commonly available polygenic score; others included type 2 diabetes and breast cancer. Reports were highly variable in terms of color, numeric risk estimate provided, categories used to describe amount of risk, and availability of additional resources and recommendations based on the test result (Fig. 2, Table 1) [21–37].

**Table 1:** Landscape of polygenic score reports

Company, country	Report(s) reviewed	Initiating stakeholder	Report medium	Eligibility criteria	Numeric risk estimate	Risk description	Colors used	Recommendations and resources^†^	Score type, No. SNPs^‡^	Sample report^‡^ Score/product development
*Clinical*										
Ambry Genetics, USA	Breast cancer	Clinician	PDF supplement to clinical report	1. Female biological sex 2. 18–84 years old 3. Non-Ashkenazi Jewish, Northern European ancestry 4. No personal history of cancer (excluding non-melanoma skin cancer) 5. No personal history of atypical hyperplasia or lobular carcinoma in situ (LCIS) 6. No personal or family history of a mutation in a breast cancer susceptibility gene^§^	Absolute lifetime risk (percentage)	Average/increased risk	Pink/grey	Y	LD adjustments + threshold^#^ 100 SNPs	22, 25
Ambry Genetics, USA	Prostate cancer—unaffected	Clinician	PDF supplement to clinical report	1. Male biological sex 2. 18–84 years old 3. Northern European ancestry 4. No personal or family history of a mutation in a prostate cancer susceptibility gene^Δ^	Absolute lifetime risk (percentage)	Average/increased risk	Blue/grey	Y	LD adjustments + threshold^#^ 72 SNPs	23, 26
Ambry Genetics, USA	Prostate cancer—affected	Clinician	PDF supplement to clinical report	1. Male biological sex 2. 18–84 years old 3. Northern European ancestry 4. No personal or family history of a mutation in a prostate cancer susceptibility gene^Δ^	Odds ratio	Average/increased risk	Blue/grey	Y	Pruning + Thresholding 72 SNPs	24, 26
Myriad Genetics, USA	Breast cancer	Clinician	PDF supplement to clinical report	1. Woman under age 85 2. European and Ashkenazi Jewish ancestry 3. No personal history of breast cancer, LCIS, hyperplasia, atypical hyperplasia, or a breast biopsy with unknown results 4. The woman does not have a mutation in a breast cancer gene (excluding monoallelic CHEK2) 5. The woman’s relatives have not been found to have a mutation in a high-penetrance breast cancer risk gene^◊^	Absolute lifetime risk (percentage)	Average/above average risk	Pink/grey/orange	Y	LD adjustments + threshold^#^ 86 SNPs	21, 27, 28
*Research*										
Scripps, USA	Coronary artery disease	Consumer	Direct to consumer smartphone application	None	Percentile	Low/intermediate/high genetic risk	Blue/red	Y	LD adjustments + threshold^#^ 57 SNPs	29
Color Health, USA	Coronary artery disease *	Consumer or clinician	Online consumer portal (data saved)	None	Percentile	Less/more genetic risk	Blue	Y	LD-Pred 6,630,150 SNPs	30–32
Gene Plaza, Belgium	Many (selected: diabetes diagnosed by a doctor)	Consumer	Website	None	None	Highly below average/below average/average/above average/highly above average	Green	N	Not publicly reported	33
Impute.me, Denmark	Many (selected: coronary artery disease)	Consumer	Website	None	Z-score	Varied e.g. “This is a lower score than the average person”	Purple	N	"Top SNP" 75,028 SNPs	34, 35
23andMe, USA	Many (selected: coronary artery disease)	Consumer	Online consumer portal (data saved)	None^➢^	Absolute risk (%) until age 80	Typical likelihood/increased likelihood	Multi	Y	LD adjustments + threshold^#^ > 2400 SNPs	36, 37

^†^Recommendations & Resources: ‘Y’ if the report included at least one statement describing medical recommendations or resources

^‡^Sample report is the most current version (March 2021) which may not be identical to the report reviewed during the study

^#^LD adjustments + threshold included both pruning and clumping LD adjustment approaches

^§^*ATM, BARD1* (if tested), *BLM* (if tested), *BRCA1, BRCA2, BRIP1, CDH1, CHEK2, FANCC* (if tested), *NBN, NF1, PALB2, PTEN, RAD51C, RAD51D, STK11* (if tested), *TP53*

^Δ^*ATM, BRCA1, BRCA2, CHEK2, EPCAM, HOXB13, MLH1, MSH2, MSH6, NBN, PALB2, PMS2, RAD51D, TP53*

^◊^High-penetrance breast cancer risk genes: *BRCA1, BRCA2, CDH1, PALB2, PTEN, STK11, TP53*, ATM c.7271 T > G., and bi-allelic *CHEK2*

^*^Color CAD report was available through a research study, which has now closed

^➢^ ‘Relevant ethnicities’ are described. However, reports are not restricted to individuals of these ancestries

^¥^Now available to women of all ancestries

Three companies (Ambry Genetics, Myriad Genetics, 23andMe) provided at least one report that integrated a polygenic risk score with other clinical risk factors to generate an absolute value of risk, displayed as a percentage [27]. Two companies (Ambry Genetics and Myriad Genetics) required a clinician to order the polygenic score. At the time of this review, Myriad and Ambry Genetics noted that their polygenic score was only available to individuals of ‘European ancestry’ (Table 1); an approach that is inconsistent with other clinical genetic tests. Given these concerns, Ambry Genetics no longer offers a polygenic score assessment as part of their clinical product (personal communication with Ambry Genetics). However, Myriad Genetics has since launched a polygenic breast cancer risk assessment score validated for women of all ancestries. Of note, both Myriad Genetics and Ambry Genetics stated that the information provided on the report may be used to ‘better guide your patient’s medical management’ or ‘to assist in the development of a treatment plan’ [22, 27]. All other reports were intended to be used only for research purposes, not to guide clinical management.

Five groups allowed for a consumer-initiated assessment without the involvement of an individual’s healthcare provider. These consumer-initiated polygenic score assessments were calculated based on either genotype or sequencing data generated from a new saliva sample sent in by the consumer or raw genotype data shared directly with the company by the consumer. Results from consumer-initiated assessments were communicated via consumer-facing online portals or a phone application.

### Development of a draft polygenic score report

**Fig. 3:**
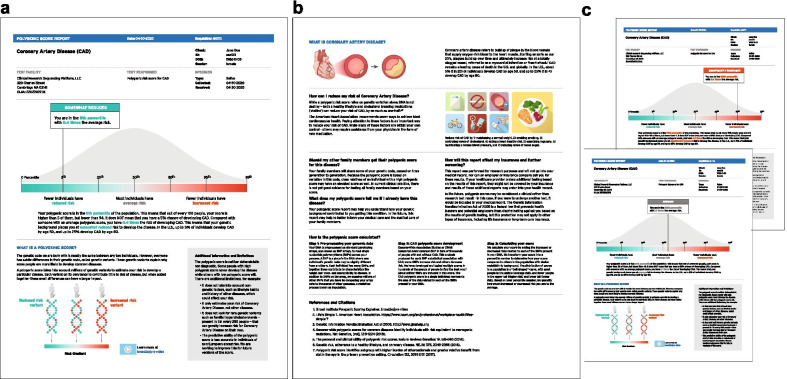
Mock polygenic score reports for coronary artery disease. Mock reports consisted of five sections: (1) Participant information, (2) Participant score, (3) ‘What is a polygenic score?’ (4) ‘What is coronary artery disease?’ and (5) ‘How can I reduce my risk of coronary artery disease?’ **a** Page one of 5th percentile (significantly reduced risk) mock report. **b** Page two of all reports. **c** Page one of 95th percentile (significantly increased risk) and 56th percentile (average risk) mock reports

We developed a two-page draft polygenic score report for CAD that was designed to be understandable by both prospective patients and clinicians (Fig. 3). To develop our report, we reviewed the design choices of existing reports, including chart types used to convey risk, descriptions of risk and labeling, color scales, and resources provided. The primary design principles that we used to highlight important concepts and optimize understandability of the report were repetition and emphasis. We also aimed to maximize accessibility and understanding of the information included in the report by minimizing technical language and using simple sentence structure [5, 6, 38].

The report consisted of five sections, each intended to explain a major concept to support polygenic score disclosure and contextualization, aided by visual representations: (1) Participant information, (2) Participant score, (3) ‘What is a polygenic score?’ (4) ‘What is coronary artery disease?’, and (5) ‘How can I reduce my risk of coronary artery disease?’. Additionally, the report included a 'Frequently Asked Questions' section to address common questions, provide a technical explanation of the test, and link to external resources.

In existing reports, color was often used to distinguish average risk (grey) from an individual’s risk (color). However, many reports used multiple color scales, which may lead to confusing hierarchies and reduced effectiveness of this element. We chose color as the main visual element to describe risk direction, using a scale from green, to grey, to red. This color scale was intended to leverage cultural associations of go-stop and cool-warm, with the grey serving as a neutral tone to represent average risk as the baseline for comparison. This color gradient was used across all risk-related visuals to bridge the relationship between an individual's polygenic score with the genetic component from which it comes [39].

A risk score ‘flag’ containing the individual’s risk category, percentile, and odds ratio was positioned on the scale from significantly reduced risk (left) to significantly increased risk (right). The flag highlighted the risk category and was displayed prominently with a solid color-coded background of green, grey, or red. The body of the flag incorporated the same color-coding in the percentile and odds ratio text, calling attention to these values and reinforcing their association to risk direction. For this coronary artery disease polygenic score report, the working group chose to incorporate the following word-based descriptions to categorize risk: ‘significantly reduced risk’ (0–5th percentile), ‘somewhat reduced risk’ (6th–19th percentile), ‘average risk’ (20th–79th percentile), ‘somewhat increased risk’ (80th–94th percentile), and ‘significantly increased risk’ (95th–99th percentile). These categories were defined based on review of literature [30].

The population distribution graph (“bell curve”) behind the risk score ‘flag’ was included to support percentile understanding, emphasizing where most people fall on the polygenic score. Bell curves, displayed in neutral colors, have been used in previous reports emphasizing the polygenic score as a percentile (Fig. 2). Labels were also placed below the distribution to further describe how many people were in each risk category. The risk gradient shared the same x-axis as the population distribution and percentile markers for consistency and repetition.

We included a textual description of the individual’s polygenic score and an explanation of their percentile below the graph. The same color-coding was applied to words pertaining to percentile, odds ratio, and risk category for further repetition and consistency. We also included statistics on coronary artery disease prevalence to provide the reader with additional context about their risk.

One unique aspect of coronary artery disease is that regardless of an individual’s genetic background, a healthy lifestyle is associated with reduced risk of the disease [38]. To communicate positive lifestyle changes that individuals can undergo to mitigate an increased genetic risk, we used simple graphics to describe seven ideal cardiovascular health measures described by the American Heart Association as Life’s Simple 7: managing blood pressure, controlling cholesterol, reducing blood sugar, exercising regularly, following a heart-healthy diet, maintaining normal weight, and stopping smoking [40]. The number of those metrics is a strong predictor of cardiovascular and all-cause mortality [30, 41].

### User experience of polygenic score risk disclosure tool

Ten participants were recruited through UserInterviews.com, an online recruitment and scheduling platform using quota sampling to enroll a relatively equal number of individuals by sex, age, and education. Individuals were required to be 18 years of age or older, speak English fluently, and be residents of the United States since disease prevalence described in the report was specific to the United States population. Participants were excluded if they worked in a genetics-related field—where prior exposure to polygenic score concepts might have influenced interpretation. Given that the score report was tailored to individuals without existing disease—e.g., including sections such as ‘How can I reduce my risk of coronary artery disease?’—those with existing CAD were also excluded.

We developed an interview guide consisting of 36 questions and prompts (Additional file 1) based on preliminary feedback from the working group’s colleagues, acquaintances, and family members. Preliminary feedback from these stakeholders about overall perception and points of confusion about the report was provided verbally in monthly meetings to three members of the research team (DGB, LP, TV).

All participants provided consent to participate in the study, which involved both audio and video recording. Interviews were conducted by a visual designer (LP) trained in user experience research and a genetic counselor (DGB). Only the two facilitators and one participant were present for each session. Interviews were conducted virtually over Zoom video conferencing and lasted one hour each. At the start of each session, participants were allowed up to fifteen minutes to independently review a mock polygenic score report for coronary artery disease indicating risk in either the 95th percentile (n = 4), 56th percentile (n = 3) or 5th percentile (n = 3). The facilitators then asked the participant to describe their experience viewing the report while being prompted to answer questions using the interview guide.

Each interview was recorded, manually transcribed by a member of the research team (DGB or TV), coded (DGB or LP) and analyzed by the facilitators using thematic analysis (DGB, LP) [42]. Each transcript was coded by DB or LP and reviewed by the other team member for agreement; conflicts were resolved through discussion (DGB, LP). Recurrent themes were identified from the data, coded, and summarized (DGB, LP). This study was approved by the Mass General Brigham Institutional Review Board (2020P003088).

## Results

### User experience testing to optimizing a polygenic score risk disclosure tool for coronary artery disease

Interviews were conducted with ten participants (5 females, mean age 50.3 years, SD = 14.8, 7 without any prior genetic testing experience) (Table 2). Although reports reviewed by study participants were based on mock data, all (10/10) participants expressed interest in receiving this report in the future based on their own genomic data.

**Table 2:** Demographics of user experience testing participants (n = 10)

Characteristics	Participants (N = 10)
Average age in years (range)	50.3 (27–70)
Female, n (%)	5 (50)
Self-reported race/ethnicity, n (%)	
White	4 (40)
Black	3 (30)
Hispanic or Latino	2 (20)
Asian	1 (10)
Educational exposure, n (%)	
Finished high school	1 (10)
Some college	4 (40)
Undergraduate degree	2 (20)
Postgraduate degree	3 (30)
Experience with genetic testing, n (%)	
Yes	3 (30)
No	7 (70)
Job	Building Design and Construction, Receptionist, Attorney, Lecturer, Freelance Development, Retired, Organizer, Analyst, Exam Proctor, ‘not reported’
Location	California, California, California, Florida, Georgia, Georgia, Massachusetts, Massachusetts, New York, New York, New York, Oregon

We identified three themes from coded transcripts: (1) Visual elements, such as color and simple graphics, enable participants to interpret, relate to, and contextualize their polygenic score, (2) Word-based descriptions of risk and polygenic scores presented as percentiles were most often recognized and understood by participants, (3) Participants had varying levels of interest in understanding complex medical and genomic information and therefore would benefit from resources that can adapt to their individual needs in real time.

**Theme One: Visual elements, such as color and simple graphics, enable participants to interpret, relate to, and contextualize their polygenic score.**

1. Color

**Fig. 4:**
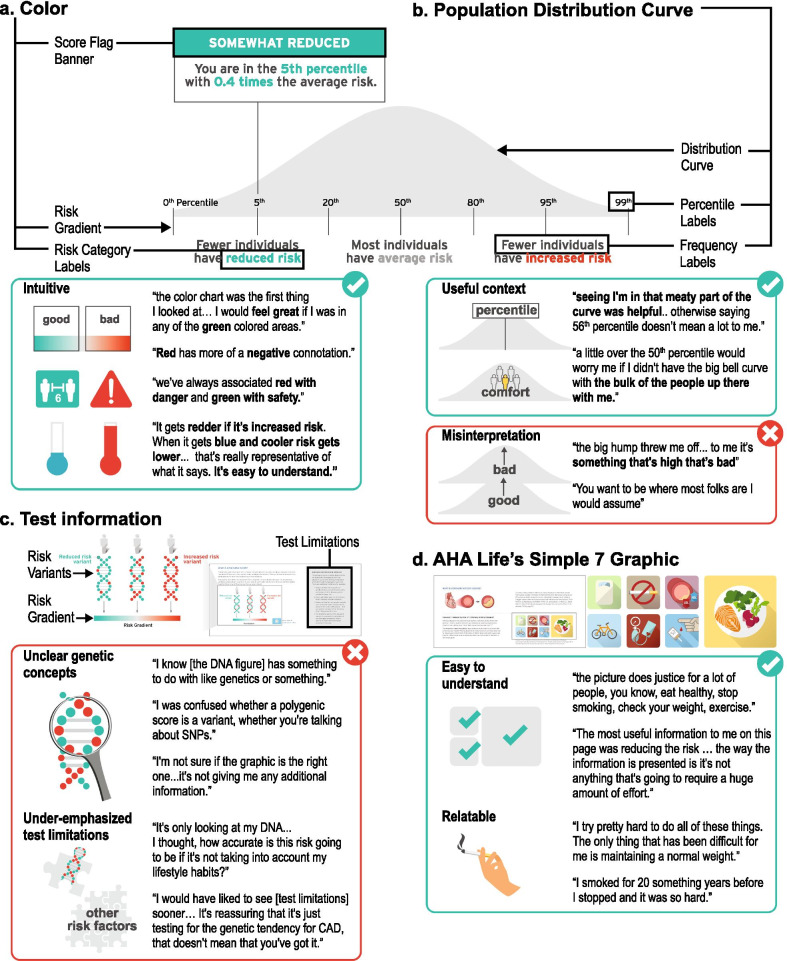
User experience testing results: Theme one. Visual elements, such as color and simple graphics, enable participants to interpret, relate to, and contextualize their polygenic score. **a** Color was the predominant design element that influenced participants’ level of concern about their hypothetical genetic risk. **b** Participants expressed differences in their understanding of the population distribution curve, interpretation of the underlying data, and association to its meaning. **c** Participants were often unclear on genetic concepts and felt that test limitations were underemphasized. **d** Participants found the cardiology and lifestyle graphics to be recognizable, relatable, and helpful for understanding the topic of the risk disclosure tool

Color was the predominant design element that influenced participants’ level of concern about their hypothetical genetic risk. As intended, participants frequently referenced these color ‘zones’ in terms of bad/good, safety/danger, unanimously considering them to be intuitive and discussing their score in terms of their position on the color gradient (Fig. 4a).

I started with the chart, um, the color chart there, that was the first thing I looked at… So, looking at a score I would feel obviously great if I was in any of the green colored areas. (Participant 3)

… we’ve always associated red with danger and green with safety. (Participant 10)

… it gets redder, if it’s increased risk, I think that's pretty good. … When it gets blue [sic] and cooler, your risk gets lower down at the end, and it says, ‘reduced risk’… It's easy to understand. (Participant 6)

2. Population distribution curve

Participants expressed differences in their understanding of the population distribution curve, interpretation of the underlying data, and association to its meaning (Fig. 4b). One participant (9), who reported an undergraduate education level, was an outlier in their misinterpretation of the curve. This participant misinterpreted the height of the curve to represent the amount of risk, associating up with more risk/bad and down with less risk/good.

The big hump kind of threw me off a little bit… to me it’s something that's high that’s bad. (Participant 9)

However, other participants reported the curve on the risk disclosure tool to be helpful in their ability to interpret their score.

It looks like most people are in the average section, like in the middle, like that’s why the curve is bigger there, cuz there are more people between the 20th and 80th percentile and then there's less people with reduced risk and less people with increased risk based on their genetics. (Participant 1)

The easiest to understand part of it is just the graphic itself… seeing I'm in that meaty part of the curve which generally, you know, says average and so I think that was helpful. … otherwise saying just someone's in, you know, 56th percentile doesn’t really mean a lot to me. (Participant 3)

Some participants even found comfort in knowing how many other people were in the same group as them.

…even just being a little over the 50th percentile would worry me more if I didn't have the big bell curve up there with the bulk of the people being up there with me. …I really like being in the middle. (Participant 6)

Most individuals have average risk, so if I’m going to be in the minority that has reduced risk, I’m good with that. (Participant 4)

3. DNA figure to convey test information and limitations

Participants’ recognition of the DNA figure often did not provide additional context for understanding polygenic risk variants (Fig. 4c).

I'm not sure if the [DNA] graphic is the right one. … it's not giving me any additional information … it's just a different variance of the first graph. (Participant 7)

Other participants who were already familiar with genetic concepts found the graphic and supporting text to be useful, but still could not fully understand the concept of polygenic inheritance.

I love explanations of how things work…it doesn't mention SNPs here anywhere and I wonder if these were actually talking about SNPs, so I was confused whether a polygenic score is actually just a variant, whether you're talking about SNPs. (Participant 6)

Regardless of whether participants understood the concepts being portrayed, they used color association to determine which individual they would be in the graphic based on their score.

I really did not understand the picture if I’m being super honest. I understand as far as the color coordination from the top to the bottom the green, the gray, and the red. But I really didn't understand…I don't look at this and think DNA. (Participant 9)

However, it was the accompanying text about test limitations, shaded in grey, that enabled most participants to grasp the authors’ intended message for this section: this test only considers polygenic contributors to disease.

I thought [the grey ‘limitations’ box] was very informative actually and it gives you a heads up that this [test] is not 100% guaranteed. It’s not taking into account if you’re a heavy drinker or heavy smoker or exerciser. (Participant 2)

The test is only going to test you for coronary artery disease so any past problems that you have or that you have at the moment is not going to affect the test I guess. (Participant 5)

Importantly, one participant did not understand that this risk estimate did not consider lifestyle and past medical history.

The information that I provided about my lifestyle and my personal statistics put me in the average range…. I would think that to get this [risk] information you would have to ask me a list of questions as far as my exercise activities, my past previous health scares, or surgeries. (Participant 9)

Another participant noted that this section about limitations “cast a little doubt” about the utility of the test.

It's only looking at my DNA, it's not considering [lifestyle habits]. I thought, how accurate is this risk going to be if it's not taking into account my lifestyle habits? (Participant 3)

To be transparent about the current limitations of polygenic scores for individuals of non-European ancestry, we included the following statement: ‘The predictive ability of the polygenic score is less accurate in individuals of non-European ancestries. We are working to improve this for future versions of the score.’ One participant who self-identified as Black expressed their feelings about this limitation, and recommended the following:

Go ahead, roll [the polygenic score] out [to everyone] and just let the non-European ancestry [individuals] know it’s going to be a little wrong, plus or minus whatever variance… It's probably still worth rolling it out to everyone. (Participant 7)

A second participant who self-identified as Black also appreciated knowing this information and suggested that everyone be informed about this limitation before pursuing the test. Interestingly, this participant stated:

Put [the limitation about race, ethnicity, ancestry] on the reports that it pertains to… so if it does not pertain to your history that wouldn't be any confusion. So, if in the ethnicity part, when you’re doing the questionnaire, provide this information just to those that it pertains to. (Participant 9)

4. Cardiology and lifestyle graphics

In general, participants found the cardiology and lifestyle graphics to be recognizable, relatable, and helpful for understanding the topic of the risk disclosure tool (Fig. 4d). When prompted to comment on their perception of the simple and familiar graphics of healthy lifestyle factors outlined by the American Heart Association, many participants took the opportunity to discuss their family medical history and their personal experience with these healthy lifestyle choices.

I try pretty hard to do all of these things. The only thing that over my life has been difficult for me is maintaining a normal weight. But all the others I've been pretty good at. (Participant 10)

… the picture does justice for a lot of people, you know, eat healthy, stop smoking, check your weight, exercise… My dad was 51 when he passed [from a heart attack] and as I get closer to that age, I get more worried … I smoked for 20 something years before I stopped and it was so hard… you know, do I want to see my kids grow up?… And I did quit smoking. (Participant 5)

Additionally, participants felt that the lifestyle recommendations represented by these images felt manageable in terms of simplicity and cost.

The most useful information to me on this page was reducing the risk … the way the information is presented is it’s not anything that's going to require a huge amount of effort. … Eating better, doing some exercise, watching your cholesterol, all that stuff just seems doable, and I think the way this information is presented here is like, oh maybe you do have this high-risk that you saw or high-risk score, but, like, here are some great ways to help offset [the risk] I guess. (Participant 3)

… it [lifestyle changes] seems easy, like you don’t have to spend thousands of dollars on, you can actually do it on your own. And it’s affordable. (Participant 2)

**Theme Two: Word-based descriptions of risk and polygenic scores presented as percentiles were most often recognized and understood by participants.**

**Fig. 5:**
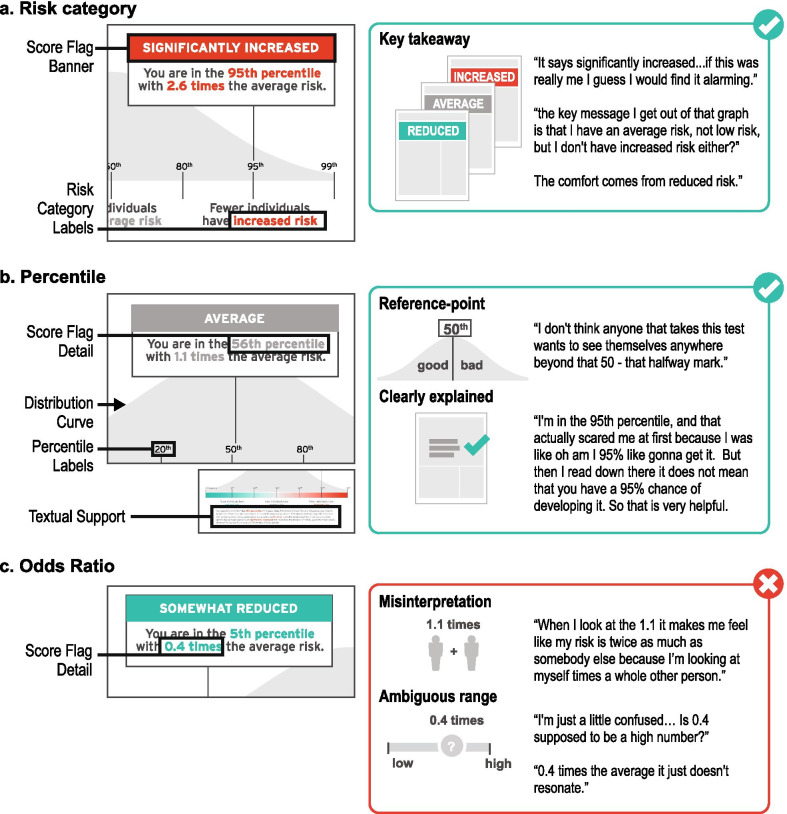
User experience testing results: Theme two. Word-based descriptions of risk and polygenic scores presented as a percentile were most often recognized and understood by participants. **a** ‘Risk category’ is an interpretation of the numeric polygenic risk estimate. **b** ‘Percentile’ is a polygenic risk estimate—on a scale from 0 to 100—describing a participant’s location in a normal distribution. **c** ‘Odds ratio’ is an estimate of risk that conveys magnitude of risk compared to ‘average risk’ of 1.0

Word-based descriptions of risk that provide high level explanations were frequently used by participants to summarize their takeaways from the risk disclosure tool (Fig. 5).

So, the key message I get out of that graph is that I have an average risk, I'm not low risk, but I don't have an increased risk either? (Participant 3)

Importantly, this risk description was often a cause for concern or comfort for some participants.

It says significantly increased… if this was really me, I guess I would find it alarming. (Participant 10)

The comfort comes from the reduced risk for me. (Participant 4)

When asked about information they found challenging in the ‘My Score’ section, many participants did not understand the odds ratio.

When I look at the 1.1 it makes me feel like my risk is twice as much as somebody else because I’m looking at myself times a whole other person. (Participant 9)

0.4 times the average it just doesn't resonate really, but you know, having a reduced risk, even if you're measured in the 5th percentile, that’s okay. (Participant 4)

Some participants’ confusion surrounding odds ratio was rooted in not having a reference point for a ‘high’ and ‘low’ odds ratio.

I'm just a little confused… Is 0.4 supposed to be a high number? (Participant 5)

I was wondering why 2.6 times the risk instead of another number, like it's really exact and I was just wondering how that came about. (Participant 1)

In contrast to an odds ratio with an ambiguous range of possible results, participants often discussed their familiarity with percentiles and commonly used percentile as the key reference point to interpret their score.

I don't think anyone that takes this test wants to see themselves anywhere beyond that 50 - that halfway mark… (Participant 7)

… it says here that out of a hundred people your score is higher than 5 but lower than 94 which I mean is simple math. (Participant 5)

Additionally, one participant acknowledged that neither number was most effective at communicating theoretical risk; rather, visual representations provided meaningful context for understanding all reported numerical values.

You're [in] the 95 percentile sounds significant… 2.6 times the average risk does not seem to be… the actual graph drives it home more so than the actual stats. (Participant 7)

**Theme Three: Participants had varying levels of interest in understanding complex medical and genomic information and therefore would benefit from resources that can adapt to their individual needs in real time.**

Universally, participants expressed their preference for receiving medical reports in simple terms with clear interpretation of results.

… [medical reports] can become overwhelming and you know for me, I want you to get to the point, where do I stand, and how did you come up with this information, and how do I relate to everybody else in my class, and some of the things that I can possibly do. (Participant 10)

**Fig. 6:**
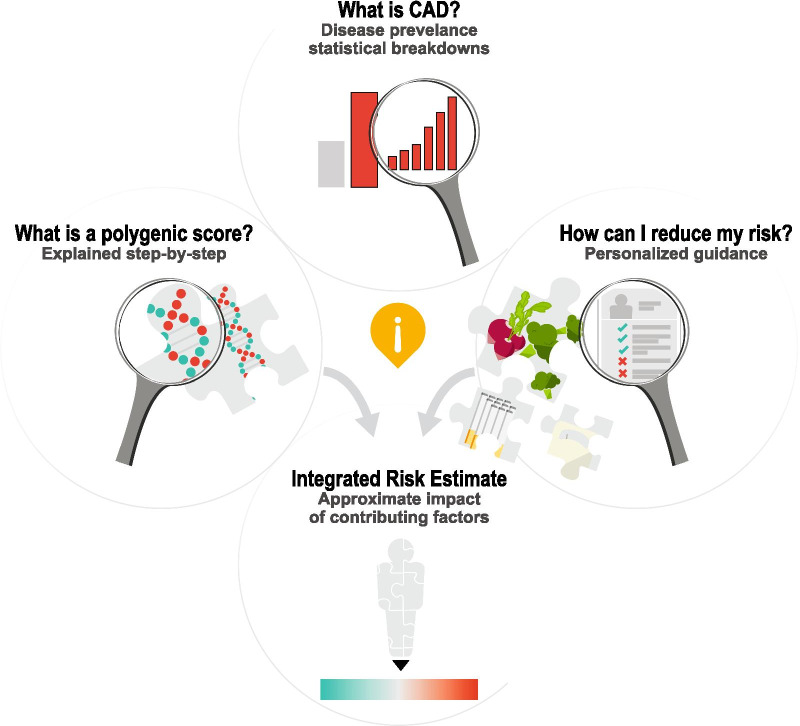
User experience testing results: Theme three. Participants had varying levels of interest in understanding complex medical and genomic information and therefore would benefit from resources that can adapt to their individual needs in real time. Participants were interested in receiving further information to answer the following questions: (1) What is a polygenic score? (2) What is CAD? (3) What is my overall risk when all contributing risk factors are considered? and (4) How can I reduce my risk?

Nevertheless, participants had different preferences for the depth of information that they desired in this tool (Fig. 6)*;* Participant 6 articulated that “it would be great if you got a choice between” the amount of information you receive*.* For example, some participants appreciated seeing a simple statistic for coronary artery disease prevalence a﻿nd shared that they were interested in more information about disease prevalence to personalize their risk.

It said 1 in 20 [people will develop coronary artery disease] when you're young, and then goes up to 1 and 4 when you're like 80. But [I would like to see] more statistics about when you're 65 or 70 broken down more. (Participant 6)

Other participants expressed confusion about the stated prevalence estimate, suggesting that additional explanation of disease prevalence, perhaps in a different format, is critical to minimize confusion and reduce the number of calculations on the individual's part.

… depending on what your age is now, [your risk to develop coronary artery disease] could be from 1 in 5 [to] 1 in 25. So where do I fit in there? … I just dismiss that part okay, but … some people might just look at it and be like oh my God, I have a 25% chance … (Participant 9)

Another topic for which participants had variable interest in receiving more information was the ‘how can I reduce my risk?’ section. While some participants were satisfied with the amount of information in this section, many participants, particularly those that received the ‘significantly increased risk’ mock score report, communicated that they would like additional guidance on how to adopt healthy lifestyle factors.

This report isn't going to detail a diet plan of course for anyone or an exercise plan for anyone, but I think there's room for more information concerning a healthy lifestyle if they fall into a higher risk category. … I think a lot of folks just don't know where to start. (Participant 7)

I would love some dietary [suggestions] on how to reduce your cholesterol… some of these are complicated issues for people to deal with and giving some ideas on how to actually do proactive things would be really good. (Participant 6)

Other participants relayed that their interest in engaging with the risk disclosure tool may depend on the result. For example, one participant stated, “if I was low, I probably wouldn't have read the whole thing.” (Participant 1) Another participant suggested that some information reminded them of ‘medication inserts’, “not very many people actually read those, but some people do. … you can ignore it if you want to, or you can go seek it out.” (Participant 3) This raises the question of how best to tailor an individual's experience viewing a genomic risk disclosure tool based on their individual result.

Some participants also indicated that other media, such as a website or a video, would be helpful for learning more about their score.

… a couple videos that explain … What is coronary artery disease? What is the score? How do we calculate it? … Even if it's just kind of a summary of the words that are there, I think having someone tell it to you is a lot easier than trying to read it and comprehend it. (Participant 3)

I wouldn't mind seeing like a little video clip of you know maybe a grandma and a granddaughter going to get this test done … you know actually see that it's real live family members taking this test, and if it seems like it's good results for them, then they could probably be good results for me… (Participant 2)

Participants also expressed their interest in learning more about how genetics and lifestyle choices together influence overall risk for coronary artery disease.

…for example, out of 100 people, 80 of them who maintained the normal weight, avoided smoking, ate healthy, exercised, reduced sugar, they [saw] results in a matter of 30 days or 2 months, something of that nature. [This fact] makes it seem easy, like you don’t have to spend thousands of dollars on [them], you can actually do it on your own. And it’s affordable. (Participant 2)

Others described their interest in seeing a personalized overall risk estimate based on genetics and lifestyle factors.

…Here is your risk score, and here is this other graphic that shows you can bring your risk down by 10 points or whatever if you were to exercise, and another three points if you were to eat right. (Participant 3)

I specifically would be able to see the effects that my actions are having, because right now I can read this and it says that if I do these things my risk will be lower, but like how much lower? Is it worth me exercising, changing my diet, quitting smoking if in all actuality it's only going to reduce my risk like 1 percentile? Or reducing it to whatever… I’m just saying: is all that effort worth it? (Participant 3)

Although not all participants felt that incorporating concrete numbers was critical for an interactive tool that layers lifestyle choices on genetic background. Rather, a direction and a broad estimate of magnitude would be sufficient.

If you were to say okay, ‘do these 5 things in your risk goes from 56th to 35th percentile’… I don't know what that means really. But if you were to say, ‘you're going to go from this average risk to this, like, reduced risk, but your reduced risk is still at the higher end of the reduced’—then, yeah, I can get that a lot more. (Participant 3)

It was rare for participants to be completely satisfied with the information included in the score report. Even though one participant stated that he received all the information he “need[ed] to know about polygenic scores in general”, he still wanted more information about how valid and trustworthy the test is, especially “if other people or other agencies or organizations have validated this particular test.” (Participant 10).

At the end of the user experience session, participants were asked a series of questions about how interested they would be in receiving a report such as this one and how comfortable they would feel viewing this information on their own. All participants were interested in receiving a polygenic score for coronary artery disease, particularly those with a family history. Additionally, all participants indicated that they would be okay reviewing their polygenic score for coronary artery disease on their own; although, five stated that they would want to discuss it with their doctor later for clarification and personalization of the information.

I wouldn’t mind receiving it on my own going through it and then maybe printing it out and then my doctor going through it with me because there’s gonna be some big words there my doctor knows, you know break it down and let’s work up with a plan and how I can avoid or lessen or stop if possible, whatever it is that’s negative like CAD. (Participant 2)

As one participant highlighted, “a lot of people don't go [to the doctor] often. Like twice a year. Maybe even once a year.” (Participant 6) For these individuals, it will be crucial for companies and researchers to consider this as they develop polygenic score risk disclosure tools that are made available directly to patients and consumers.

### Modifying the report based on user feedback

Although color was well-understood by participants, we modified colors used to convey risk throughout the report to reduce the strength of association. The red–orange color, which was used to convey high risk, was altered to add cooler undertones; the green color, which was used to convey reduced risk, was changed to a blue/teal. In addition, we removed the polygenic score odds ratio from the risk score 'flag' for two reasons—first, the score percentile was best understood by most users; and second, the relative risk for a given percentile are known to vary according to genetic ancestry. Revised sample reports – significantly increased risk, average risk, and significantly decreased risk – are available as supplementary files (Additional file 2). Last, we created a video explainer to provide additional education and resources for users to learn more about polygenic scores [43].

## Discussion

In this qualitative user experience study, we describe a generalizable process for designing a polygenic score report for a complex disease. Our findings highlight two key insights in genomic implementation of risk disclosure using polygenic scores. These findings may inform the return of polygenic scores through commercial entities and research initiatives, such as the United Kingdom Accelerating Detection of Disease Challenge and eMERGE network [44, 45]. The vast scope of these projects will require new tools for polygenic score disclosure and education through new service delivery models that may rely on novel media for communicating with patients and consumers.

**Fig. 7:**
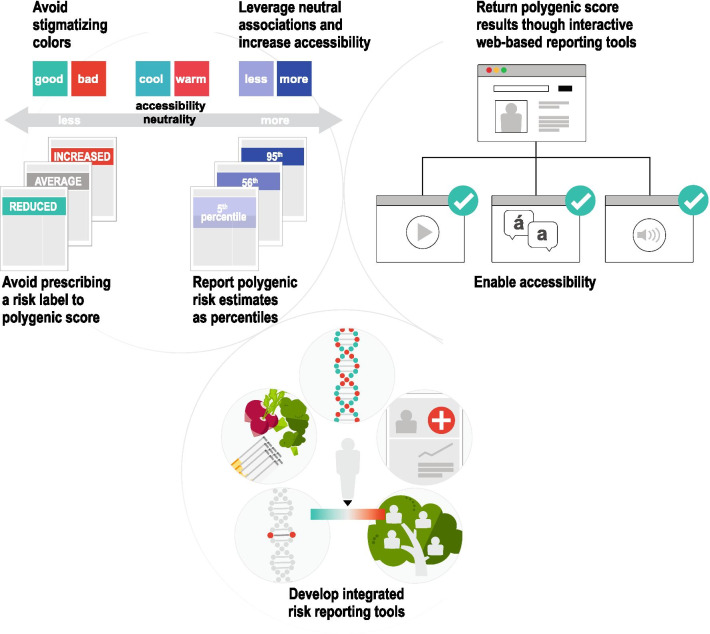
Next steps in genomic risk disclosure. The following should be considered when developing genomic risk disclosure tools: (1) Use non-stigmatizing colors that leverage neutral associations and are accessible for individuals with color blindness, (2) Report polygenic scores as percentile and avoid prescribing a categorical risk label, (3) Use interactive web-based reporting tools that enable accessibility options and personalized experiences, (4) Develop reporting tools that integrate a range of disease risk factors

First, seemingly routine design and reporting aspects of a report such as choice of color, visual and numerical estimates can have significant impact on participant understanding. For example, we noted that color, which varied across mock reports, was an important element of design that strongly influenced risk perception. The strength of participants’ red-green color association merits consideration of the intended goal for the use of color in the score report: enhancing readability, emphasizing key information, promoting perceived actionability, or sparking concern or comfort. For each of those goals, the choice of color would differ. For our study, the red-green color served strongly to spark concern or comfort. Importantly, we should be mindful of the inherent limitations of using color to convey health risks, such as the varying cultural associations of different colors, and weakened impact for individuals with color blindness (Fig. 7) [46, 47]. In future studies, it would be important to consider alternative options, such as a monochromatic color scale [48]. This could simplify the amount of visual decoding required by patients, and would lend itself better to describing less/more risk, rather than low/high risk, a subtlety that nevertheless has implications for risk perception.

In addition to color, participants often recalled percentile and ‘risk category’ as the key messages of the report. Although percentiles do not correspond to medical guidelines at this time, we chose to emphasize a percentile as the main result since it provided an unambiguous range of reference—0 to 99—that was most understood by participants. We removed the odds ratio from the report because it was poorly understood by participants and varies according to genetic ancestry [49]. Future efforts to include odds ratio or another metric of relative risk—once available with adequate precision across diverse ancestries—will likely require additional visuals or text to aid understanding. Alternatively, we note ongoing work that may allow for reporting of absolute risk estimation of a given disease informed by an individual’s polygenic score [50, 51]. In the context of this study, we felt that it was appropriate to bin and assign risk categories by percentile—such as ‘significantly increased risk’ for those with polygenic score above the 95th percentile—to evaluate perceived value and meaning by participants. However, we appreciate that such labels are inherently arbitrary for a continuous trait and the clinical utility of varying thresholds has not been well-established. We chose not to include an absolute risk estimate in our report. At this time, we recognize that polygenic scores are less precise across populations and by integrating them with existing, problematic, race-corrected clinical risk models, we risk perpetuating and exacerbating health disparities [52, 53].

Second, static genomic test reports that limit one’s ability to explore results are not ideal for polygenic risk disclosure. Risk disclosure tools that enable patients and consumers to have a personalized, interactive experience with their genomic report are likely to provide additional value (Fig. 7). One such medium to consider for this purpose is reader-driven narrative visualization, sometimes referred to as ‘scrollytelling,’ which are online, user-centric, interactive tools that enable dynamic data visualization to educate the reader about complex concepts [54]. To date, this medium has been used to educate about public health issues such as the United States measles outbreak in 2015 and the Covid-19 pandemic [55–57]. We see an unexplored opportunity to develop and evaluate the utility of reader-driven narrative visualization tools in the context of preventive genomic medicine to support personalized healthcare.

In the context of coronary artery disease, a reader-driven narrative visualization risk disclosure tool could provide patients with resources and guidance outside of physician-prescribed medications and tests. For example, some patients or consumers may benefit from additional information about how to manage one's blood sugar levels through diet that would inform subsequent shared decision making with their clinical care team. Lastly, reader-driven narrative visualization tools for polygenic score results could also facilitate expansion of accessibility options, such as providing language translation of content for non-English speakers, enabling audio playback of content for individuals with visual impairments, and tailoring content and recommendations for individuals with low health literacy (Fig. 7).

This study has several limitations. First, given the speed at which polygenic score reports are being developed and modified, existing reports may have been updated since our review. Second, we aimed to recruit a cohort of participants with diverse backgrounds (education/work experience, age, geographic location, race/ethnicity) using a national online recruitment platform. After transcribing, coding, and analyzing ten in-depth interviews, we reached thematic saturation as no new themes were observed. Although there are no published guidelines or tests of adequacy for estimating the sample size required to reach saturation’ [58], we appreciate that our sample size was small and likely does not fully reflect the potential experience of all potential users. Future larger studies should be conducted with specific focus on individuals with reduced health literacy, variable language preferences, and disabilities. Our study also excluded individuals with a history of coronary artery disease from participating in the study. Future studies of individuals with existing disease—in whom the interpretation of a genetic predisposition would be different—are warranted.

Third, this study did not evaluate the clinicians' experience viewing a polygenic score risk disclosure tool. It is important to acknowledge that clinicians may have different preferences for the design of a polygenic score risk disclosure tool. As efforts for returning polygenic scores continue to advance, it will be important to assess perceived utility of report elements by clinicians with and without clinical genetics expertise, and to standardize terminology and thresholds for risk labeling by disease area. Fourth, our study focused on return of a mock polygenic score report rather than an assessment of an individual’s actual risk.

## Conclusions

Our approach of building a multidisciplinary team to draft a polygenic score report with iterative refinement based on formal user experience testing is likely to provide a generalizable framework for disclosure of increasingly available and complex tools for ‘genome interpretation.’ Ongoing efforts by international groups are likely to further refine the set of reporting and educational tools that will best enable genetic risk assessment to improve public health.

## Supplementary Information

**Additional file 1**: Interview Guide.

**Additional file 2**: Revised polygenic score reports for coronary artery disease. A. Page one of ‘significantly increased risk’ report. B. Page one of ‘average risk’ report. C. Page one of ‘significantly decreased risk’ report. D. Page two of all reports. All reports consist of five sections: (1) Participant information, (2) Participant score, (3) ‘What is a polygenic score?’ (4) ‘What is coronary artery disease?’ and (5) ‘How can I reduce my risk of coronary artery disease?’

## Data Availability

Individual level transcripts generated and analysed during the study are not publicly available as they may provide identifying information. For additional information, please contact D.G.B. (Deanna.brockman@mgh.harvard.edu).

## Abbreviations

ACMG: American College of Medical Genetics
CAD: Coronary artery disease
DNA: Deoxyribonucleic acid

## References

[1] Lewis CM, Vassos E (2020). Polygenic risk scores: from research tools to clinical instruments. Genome Med.

[2] Wand H, Lambert SA, Tamburro C, Iacocca MA, O’Sullivan JW, Sillari C (2021). Improving reporting standards for polygenic scores in risk prediction studies. Nature.

[3] Lewis ACF, Green RC (2021). Polygenic risk scores in the clinic: new perspectives needed on familiar ethical issues. Genome Med.

[4] Haga SB, Mills R, Pollak KI, Rehder C, Buchanan AH, Lipkus IM (2014). Developing patient-friendly genetic and genomic test reports: formats to promote patient engagement and understanding. Genome Med.

[5] Williams JL, Rahm AK, Stuckey H, Green J, Feldman L, Zallen DT (2016). Enhancing genomic laboratory reports: a qualitative analysis of provider review. Am J Med Genet A.

[6] Stuckey H, Williams JL, Fan AL, Rahm AK, Green J, Feldman L (2015). Enhancing genomic laboratory reports from the patients’ view: a qualitative analysis. Am J Med Genet A.

[7] Davis KW, Erby LH, Fiallos K, Martin M, Wassman ER (2019). A comparison of genomic laboratory reports and observations that may enhance their clinical utility for providers and patients. Mol Genet Genomic Med.

[8] Davis KW, Roter DL, Schmidlen T, Scheinfeldt LB, Klein WMP. Testing a best practices risk result format to communicate genetic risks. Patient Education and Counseling [Internet]. 2020 [cited 2021 Jan 6]. http://www.sciencedirect.com/science/article/pii/S0738399120305589. 10.1016/j.pec.2020.10.021 PMC8053732 33131927

[9] Williams JL, Rahm AK, Zallen DT, Stuckey H, Fultz K, Fan AL (2018). Impact of a patient-facing enhanced genomic results report to improve understanding, engagement, and communication. J Genet Counsel.

[10] Matthijs G, Souche E, Alders M, Corveleyn A, Eck S, Feenstra I (2016). Guidelines for diagnostic next-generation sequencing. Eur J Hum Genet.

[11] Smith K, Martindale J, Wallis Y, Bown N, Leo N, Creswell L (2015). General genetic laboratory reporting recommendations.

[12] Richards S, Aziz N, Bale S, Bick D, Das S, Gastier-Foster J (2015). Standards and guidelines for the interpretation of sequence variants: a joint consensus recommendation of the American College of Medical Genetics and Genomics and the Association for Molecular Pathology. Genet Med.

[13] den Dunnen JT, Dalgleish R, Maglott DR, Hart RK, Greenblatt MS, McGowan-Jordan J (2016). HGVS recommendations for the description of sequence variants: 2016 update. Hum Mutat.

[14] Farmer GD, Gray H, Chandratillake G, Raymond FL, Freeman ALJ (2020). Recommendations for designing genetic test reports to be understood by patients and non-specialists. Eur J Hum Genet.

[15] Daly MB, Reiser G, Pal T, Kohlmann W, Senter-Jamieson L, Kurian AW, et al. NCCN guidelines index table of contents genetic/familial high-risk assessment: breast, ovarian, and pancreatic discussion. Risk Assess 2020;122.

[16] US Preventive Services Task Force (2019). Risk assessment, genetic counseling, and genetic testing for BRCA-related cancer: US preventive services task force recommendation statement. JAMA.

[17] Aragam KG, Natarajan P (2020). Polygenic scores to assess atherosclerotic cardiovascular disease risk. Circ Res.

[18] Aragam KG, Chaffin M, Hindy G, Cagan A, Weng LC, Lubitz S (2019). Clinical characteristics and treatment patterns of patients at high polygenic risk for coronary artery disease. J Am Coll Cardiol.

[19] Inouye M, Abraham G, Nelson CP, Wood AM, Sweeting MJ, Dudbridge F (2018). Genomic risk prediction of coronary artery disease in 480,000 adults. J Am Coll Cardiol.

[20] Lambert SA, Gil L, Jupp S, Ritchie S, Xu Y, Buniello A, et al. The Polygenic Score Catalog: an open database for reproducibility and systematic evaluation. medRxiv. Cold Spring Harbor Laboratory Press; 2020. 10.1038/s41588-021-00783-5 PMC11165303 33692568

[21] Gallagher S, Hughes E, Wagner S, Tshiaba P, Rosenthal E, Roa BB (2020). Association of a polygenic risk score with breast cancer among women carriers of high- and moderate-risk breast cancer genes. JAMA Netw Open.

[22] AmbryScore-Breast|Ambry Genetics [Internet]. AmbryScore for breast cancer. 2021 [cited 2021 Mar 3]. https://www.ambrygen.com/providers/ambryscore/breast; [cited 2021 Mar 4] Wayback Machine: https://web.archive.org/web/20210304005349/https://www.ambrygen.com/providers/ambryscore/breast.

[23] AmbryScore [Internet]. Personalized Prostate Cancer Risk Estimate (Unaffected). [cited 2021 Apr 13]. https://www.ambrygen.com/file/material/view/976/AmbryScore%20Sample%20Report%202-%20Unaffected%20Male%20at%20Increased%20Risk.pdf; [cited 2020 Sep 29] Wayback Machine, web.archive.org/web/20200929203659/https://www.ambrygen.com/file/material/view/976/AmbryScore%20Sample%20Report%202-%20Unaffected%20Male%20at%20Increased%20Risk.pdf.

[24] AmbryScore [Internet]. Personalized Prostate Cancer Risk Estimate (Affected). 2020 [cited 2021 Jul 20]. https://www.ambrygen.com/file/material/view/978/AmbryScore%20Sample%20Report%204%20-%20Affected%20Male%20at%20Increased%20Risk.pdf; [cited 2020 Sep 29] Wayback Machine: https://web.archive.org/web/20200929191018/https://www.ambrygen.com/file/material/view/978/AmbryScore%20Sample%20Report%204%20-%20Affected%20Male%20at%20Increased%20Risk.pdf.

[25] Black MH, Li S, Laduca H, Chen J, Hoiness R, Gutierrez S, et al. Validation of a polygenic risk score for breast cancer in unaffected Caucasian women referred for genetic testing, 4.

[26] Black MH, Li S, LaDuca H, Chen J, Hoiness R, Gutierrez S (2020). Validation of a prostate cancer polygenic risk score for clinical use. Prostate.

[27] Myriad riskScore [Internet]. Myriad myRisk. 2021 [cited 2021 Apr 13]. https://myriadmyrisk.com/riskscore/.

[28] Hughes E, Tshiaba P, Gallagher S, Wagner S, Judkins T, Roa B (2020). Development and validation of a clinical polygenic risk score to predict breast cancer risk. JCO Precis Oncol.

[29] Muse EM. Moving beyond clinical risk scores with a mobile app for the genomic risk of coronary artery disease. bioRxiv [Internet]. 2017 [cited 2021 Apr 13]; https://www.biorxiv.org/content/10.1101/101519v1.

[30] Khera AV, Chaffin M, Aragam KG, Haas ME, Roselli C, Choi SH (2018). Genome-wide polygenic scores for common diseases identify individuals with risk equivalent to monogenic mutations. Nat Genet.

[31] Homburger JR, Neben CL, Mishne G, Zhou AY, Kathiresan S, Khera AV (2019). Low coverage whole genome sequencing enables accurate assessment of common variants and calculation of genome-wide polygenic scores. Genome Med.

[32] Color Genome-Wide Polygenic Score [Internet]. 2021. https://www.color.com/wp-content/uploads/2021/03/Color-Genome-wide-Polygenic-Score.pdf.

[33] GenePlaza | App Store - Health Traits [Internet]. GenePlaza. [cited 2021 Mar 3]. https://www.geneplaza.com.

[34] Impute.me [Internet]. [cited 2021 Mar 3]. https://www.impute.me/AllDiseases/.

[35] Folkersen L, Pain O, Ingason A, Werge T, Lewis CM, Austin J (2020). Impute.me: an open-source, non-profit tool for using data from direct-to-consumer genetic testing to calculate and interpret polygenic risk scores. Front Genet.

[36] 23andMe [Internet]. Coronary artery disease (Report). [cited 2021 Apr 13]. https://medical.23andme.com/wp-content/uploads/2020/06/cad_increased.pdf.

[37] Ashenhurst JR, Zhan J, Multhaup ML, Kita R, Sazonova OV, Krock B, et al. A generalized method for the creation and evaluation of polygenic scores. 2021;20.

[38] Joseph G, Pasick RJ, Schillinger D, Luce J, Guerra C, Cheng JKY (2017). Information mismatch: cancer risk counseling with diverse underserved patients. J Genet Couns.

[39] Borkin MA, Bylinskii Z, Kim NW, Bainbridge CM, Yeh CS, Borkin D (2016). Beyond memorability: visualization recognition and recall. IEEE Trans Vis Comput Graph.

[40] Goff DC, Lloyd-Jones DM, Bennett G, Coady S, D’Agostino RB, Gibbons R (2014). 2013 ACC/AHA guideline on the assessment of cardiovascular risk: a report of the American College of Cardiology/American Heart Association task force on practice guidelines. J Am Coll Cardiol.

[41] Khera AV, Emdin CA, Drake I, Natarajan P, Bick AG, Cook NR (2016). Genetic risk, adherence to a healthy lifestyle, and coronary disease. N Engl J Med Mass Med Soc.

[42] Clarke V, Braun V. Thematic analysis. In: Encyclopedia of critical psychology. New York: Springer; 2014. p. 1947–52.

[43] New genetic test for heart attack risk launched for patients at Mass General|Broad Institute [Internet]. [cited 2021 April 11]. https://www.broadinstitute.org/news/new-genetic-test-heart-attack-risk-launched-patients-mass-general.

[44] McCarty CA, Chisholm RL, Chute CG, Kullo IJ, Jarvik GP, Larson EB (2011). The eMERGE network: a consortium of biorepositories linked to electronic medical records data for conducting genomic studies. BMC Med Genomics.

[45] The UK strategy for rare diseases: 2020 update to the implementation plan for England. 2020; p. 31.

[46] Aslam MM (2006). Are you selling the right colour? A cross-cultural review of colour as a marketing cue. J Mark Commun.

[47] Wong B (2011). Points of view: color blindness. Nat Methods.

[48] Damle A (2010). Using monochromatic design views to avoid a premature fixation on design solutions. Hum Factors.

[49] Fahed AC, Aragam KG, Hindy G, Chen Y-DI, Chaudhary K, Dobbyn A (2020). Transethnic transferability of a genome-wide polygenic score for coronary artery disease. Circ Genomic Precis Med.

[50] Weale ME, Riveros-Mckay F, Selzam S, Seth P, Moore R, Tarran WA (2021). Validation of an integrated risk tool, including polygenic risk score, for atherosclerotic cardiovascular disease in multiple ethnicities and ancestries. Am J Cardiol.

[51] Gao C, Polley EC, Hart SN, Huang H, Hu C, Gnanaolivu R, et al. Risk of breast cancer among carriers of pathogenic variants in breast cancer predisposition genes varies by polygenic risk score. J Clin Oncol. 2021;JCO2001992. 10.1200/JCO.20.01992 PMC8330969 34101481

[52] Martin AR, Kanai M, Kamatani Y, Okada Y, Neale BM, Daly MJ (2019). Current clinical use of polygenic scores will risk exacerbating health disparities. Nat Genet.

[53] Vyas DA, Eisenstein LG, Jones DS (2020). Hidden in plain sight—reconsidering the use of race correction in clinical algorithms. N Engl J Med.

[54] Lee B, Riche NH, Isenberg P, Carpendale S (2015). More than telling a story: transforming data into visually shared stories. IEEE Comput Graph Appl.

[55] Collins K, Pearce A, Armstrong D. Why measles may just be getting started. Bloomberg [Internet]. 2015 Feb 16 [cited 2021 Feb 25]. https://www.bloomberg.com/graphics/2015-measles-outbreaks/.

[56] Tierney L, Fox J, Meko T, Alcantara C, Muyskens J, Tan S, et al. More than 2,508,000 people have died from the coronavirus worldwide. The Washington Post [Internet]. 2021. https://www.washingtonpost.com/graphics/2020/world/mapping-spread-new-coronavirus/.

[57] Fox J, Shin Y, Emamdjomeh A. How epidemics like covid-19 end (and how to end them faster). 2020. https://www.washingtonpost.com/graphics/2020/health/coronavirus-how-epidemics-spread-and-end/?tid=graphics-story.

[58] Morse J, Denzin NK, Lincoln YS (1994). Designing funded qualitative research. Handbook of qualitative research.

