# Supplementary material for: Design and user experience testing of a polygenic score report: a qualitative study of prospective users
Source: BMC Med Genomics. 2021 Oct 1;14:238. doi: 10.1186/s12920-021-01056-0 (PMC8485114; doi:10.1186/s12920-021-01056-0)
Supplement: Supplementary file 2 — Additional file 2: Revised polygenic score reports for coronary artery disease. A. Page one of ‘significantly increased risk’ report. B. Page one of ‘average risk’ report. C. Page one of ‘significantly decreased risk’ report. D. Page two of all reports. All reports consist of five sections: (1) Participant information, (2) Participant score, (3) ‘What is a polygenic score?’ (4) ‘What is coronary artery disease?’ and (5) ‘How can I reduce my risk of coronary artery disease?’ [file 12920_2021_1056_MOESM2_ESM.pdf]

Coronary Artery Disease (CAD)

Coronary Artery Disease Genetic Score, Color

Date:
Name:
DOB:

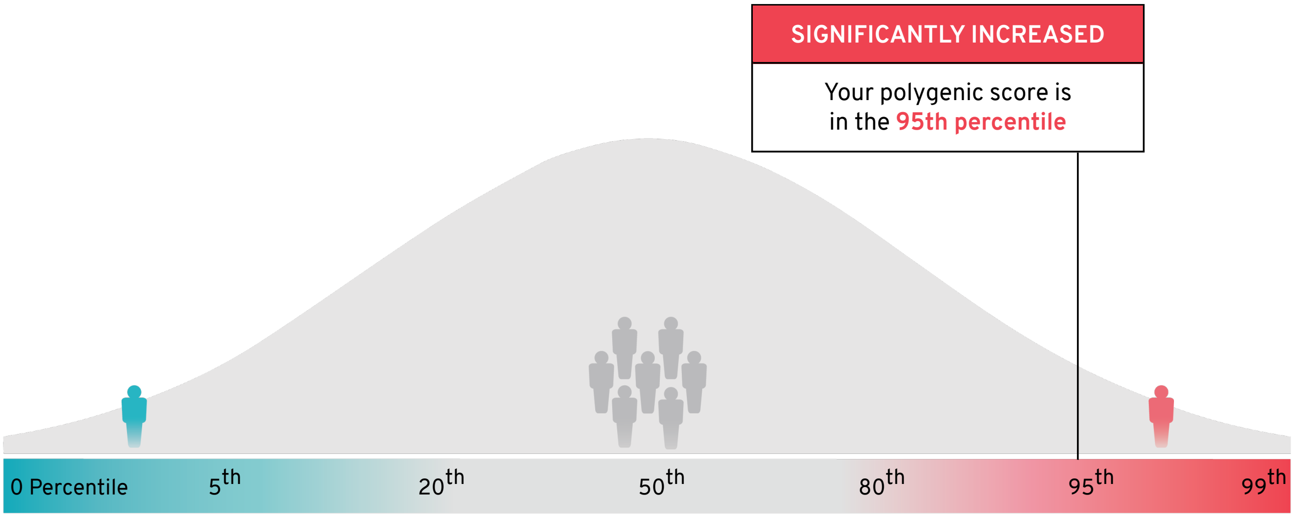

Fewer individuals have decreased risk

Most individuals have average risk

Fewer individuals have increased risk

Your polygenic score is in the 95th percentile. This does not mean that you have a 95% chance of developing coronary artery disease. Rather, it means that out of every 100 people, your polygenic score is higher than 95 people, and the same or lower than 5. This means that your genetic background places you at significantly increased risk to develop the disease. In the United States, up to 5% of individuals develop coronary artery disease by age 50, and up to 25% develop coronary artery disease by age 80.

WHAT IS A POLYGENIC SCORE?

The genetic code we are born with is mostly the same between any two individuals. However, everyone has subtle differences in their genetic code, called genetic variants. Genetic variants can explain why some people are more likely to develop common diseases.

A polygenic score takes into account millions of genetic variants to estimate your risk to develop a particular disease. Each variant on its own tends to contribute little to risk of disease, but when added together these small differences can have a larger impact.

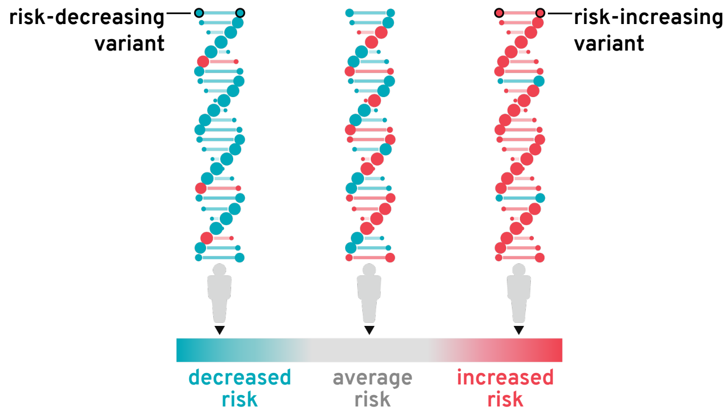

Additional information and limitations

A polygenic score is neither deterministic nor diagnostic. Some people with a high polygenic score will never develop the disease while others with a low polygenic score will.

Limitations of a polygenic score

- It does not take into account non-genetic factors, such as lifestyle habits and history of other diseases, which could affect your risk.
- It only estimates your risk of coronary artery disease, not other diseases.
- It does not look for rare genetic variants such as familial hypercholesterolemia – present in 1 in every 250 people – that can greatly increase risk for coronary artery disease on their own.
- Although the polygenic score predicts risk in all ancestries, the scores have been best validated in individuals of European ancestry.

Learn more at polygenicscores.org/explained

Coronary Artery Disease (CAD)

Coronary Artery Disease Genetic Score, Color

Date:
Name:
DOB:

AVERAGE

Your polygenic score is in the 51st percentile

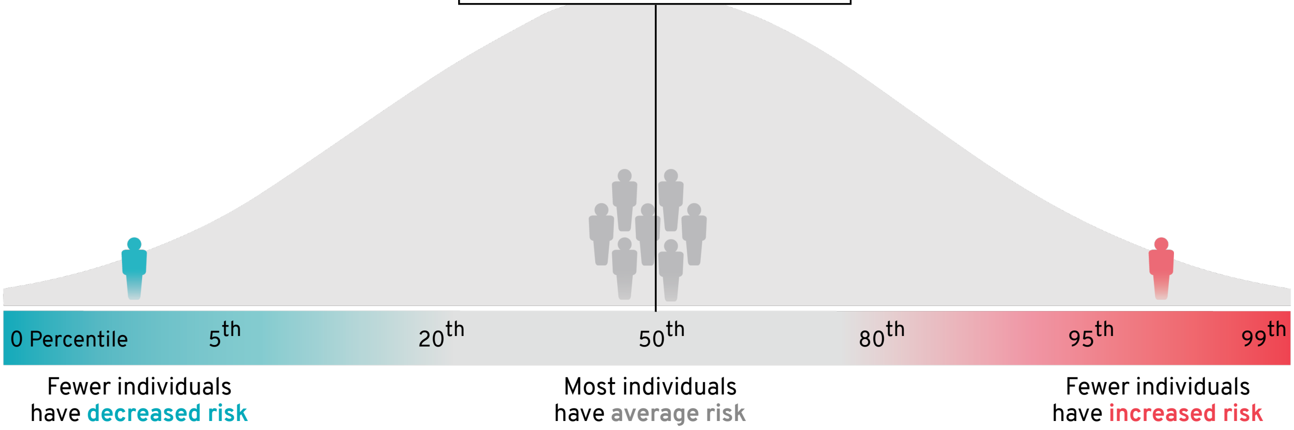

Your polygenic score is in the 51st percentile. This does not mean that you have a 51% chance of developing coronary artery disease. Rather, it means that out of every 100 people, your polygenic score is higher than 51 people, and the same or lower than 49. This means that your genetic background places you at average risk to develop the disease. In the United States, up to 5% of individuals develop coronary artery disease by age 50, and up to 25% develop coronary artery disease by age 80.

WHAT IS A POLYGENIC SCORE?

The genetic code we are born with is mostly the same between any two individuals. However, everyone has subtle differences in their genetic code, called genetic variants. Genetic variants can explain why some people are more likely to develop common diseases.

A polygenic score takes into account millions of genetic variants to estimate your risk to develop a particular disease. Each variant on its own tends to contribute little to risk of disease, but when added together these small differences can have a larger impact.

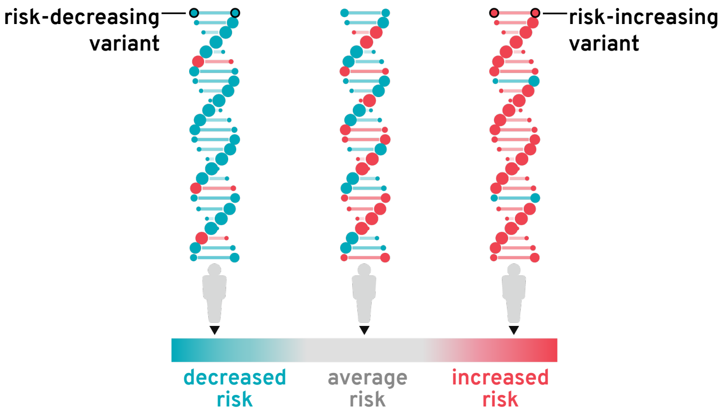

Additional information and limitations

A polygenic score is neither deterministic nor diagnostic. Some people with a high polygenic score will never develop the disease while others with a low polygenic score will.

Limitations of a polygenic score

- It does not take into account non-genetic factors, such as lifestyle habits and history of other diseases, which could affect your risk.
- It only estimates your risk of coronary artery disease, not other diseases.
- It does not look for rare genetic variants such as familial hypercholesterolemia – present in 1 in every 250 people – that can greatly increase risk for coronary artery disease on their own.
- Although the polygenic score predicts risk in all ancestries, the scores have been best validated in individuals of European ancestry.

Learn more at polygenicscores.org/explained

Coronary Artery Disease (CAD)

Coronary Artery Disease Genetic Score, Color

Date:
Name:
DOB:

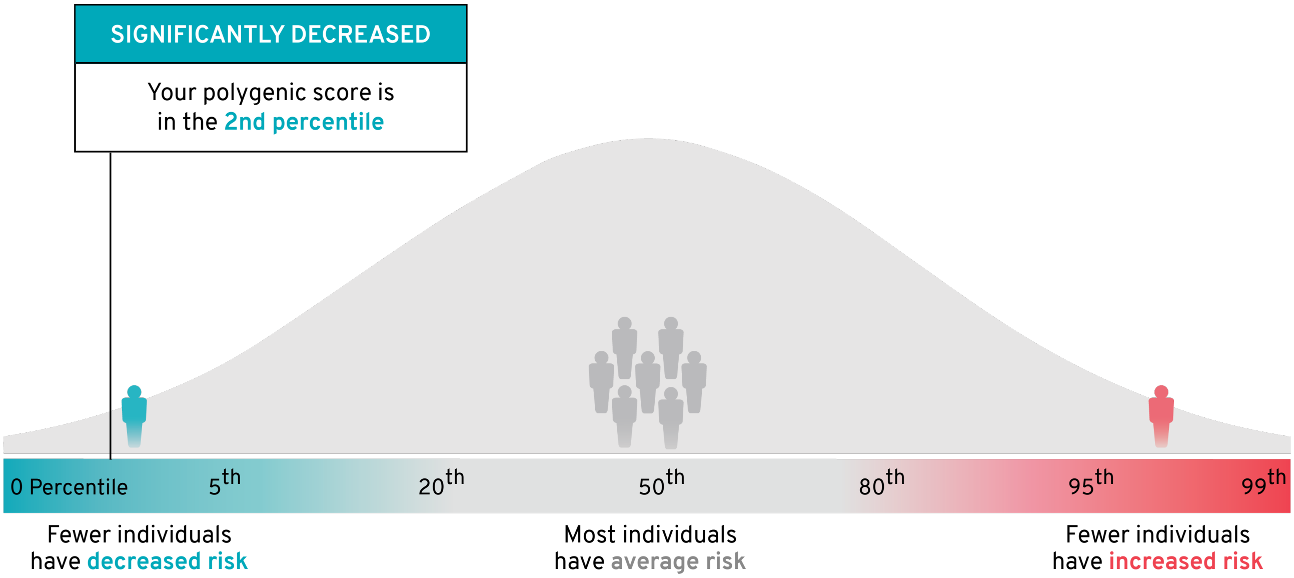

Your polygenic score is in the **2nd percentile**. This does not mean that you have a 2% chance of developing coronary artery disease. Rather, it means that out of every 100 people, your polygenic score is higher than 2 people, and the same or lower than 98. This means that your genetic background places you at **significantly decreased risk** to develop the disease. In the United States, up to 5% of individuals develop coronary artery disease by age 50, and up to 25% develop coronary artery disease by age 80.

WHAT IS A POLYGENIC SCORE?

The genetic code we are born with is mostly the same between any two individuals. However, everyone has subtle differences in their genetic code, called genetic variants. Genetic variants can explain why some people are more likely to develop common diseases.

A polygenic score takes into account millions of genetic variants to estimate your risk to develop a particular disease. Each variant on its own tends to contribute little to risk of disease, but when added together these small differences can have a larger impact.

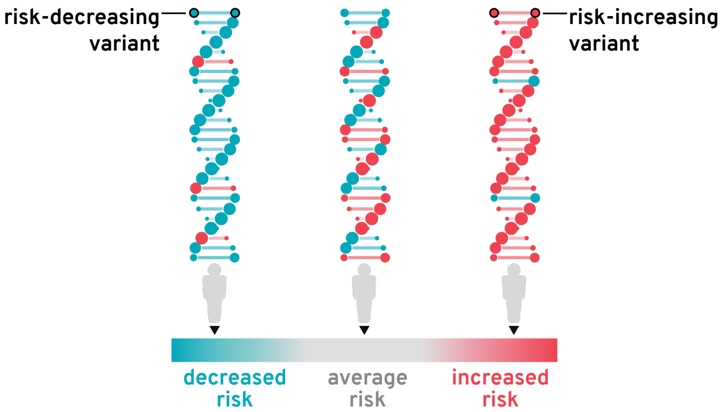

Additional information and limitations

A polygenic score is neither deterministic nor diagnostic. Some people with a high polygenic score will never develop the disease while others with a low polygenic score will.

Limitations of a polygenic score

- It does not take into account non-genetic factors, such as lifestyle habits and history of other diseases, which could affect your risk.
- It only estimates your risk of coronary artery disease, not other diseases.
- It does not look for rare genetic variants such as familial hypercholesterolemia – present in 1 in every 250 people – that can greatly increase risk for coronary artery disease on their own.
- Although the polygenic score predicts risk in all ancestries, the scores have been best validated in individuals of European ancestry.

Learn more at
polygenicscores.org/explained

WHAT IS CORONARY ARTERY DISEASE?

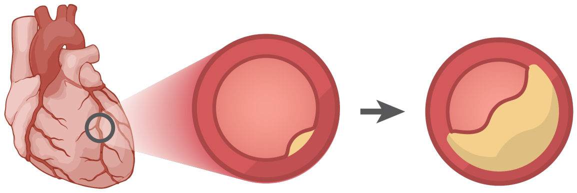

Coronary artery disease (CAD) occurs due to buildup of plaque in the blood vessels that supply oxygen-rich blood to the heart muscle, and remains a leading cause of death in the U.S. and globally. Starting as early as our 20's, plaques can build up and ultimately increase risk of a totally clogged vessel, referred to as a myocardial infarction or 'heart attack.' In the United States, about 5% (1 in 20) of individuals develop CAD by age 50, and up to 25% (1 in 4) develop CAD by age 80.

How can I decrease my risk of coronary artery disease?

A polygenic score for coronary artery disease is based only on the genetic code you were born with. Even though you can't change your polygenic score, 'DNA is not destiny' – both a healthy lifestyle and cholesterol lowering medications ('statins') can significantly decrease your risk of coronary artery disease.[1, 2]

The American Heart Association has outlined seven key factors that contribute to ideal cardiovascular health ('Life's Simple 7').[3] Individuals with at least five of these healthy factors have a 78% reduced risk for heart-related death compared to people without any healthy factors.[4]

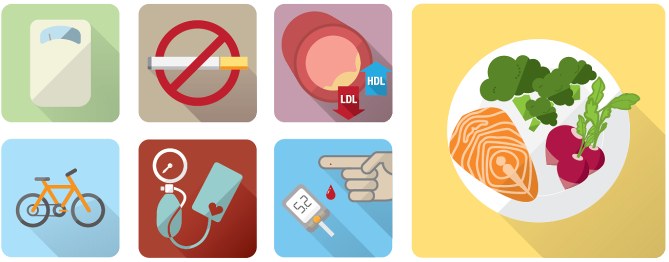

'Simple 7' factors for ideal cardiovascular health: 1) maintain a normal weight, 2) stop smoking, 3) control cholesterol levels, 4) follow a heart-healthy diet, 5) exercise regularly, 6) maintain a normal blood pressure, and 7) reduce blood sugar.

Should my other family members get their polygenic score for this disease?

Your family members will share some of your genetic code, passed on from generation to generation. Because the polygenic score is based on variation in this code, close relatives of an individual with a high polygenic score may have an elevated score as well. In current clinical practice, there is not yet good evidence for testing all family members based on your score.

What does my polygenic score tell me if I already have this disease?

Your polygenic score report may help you understand how your genetic background contributed to you developing this condition. In the future, this report may help to better inform your medical care and the medical care of your family members.

How might this information affect my insurance or employment?

The Genetic Information Nondiscrimination Act of 2008 is a federal law that prevents health insurers and potential employers from discriminating against you based on genetic test results.[5] However, this protection may not apply to other types of insurance, including life insurance or long-term care insurance.

How might these results affect my medical care?

Based on the results of this test, your healthcare provider may suggest additional screening, testing, or medications to reduce your risk of coronary artery disease. We recommend that you share and discuss these results with your doctor.

How is the polygenic score calculated?

Step 1: Pre-processing your genomic data
Your DNA was analyzed using low-coverage genome sequencing (lcWGS)[6] to read single nucleotide polymorphisms (SNPs) across your genome. A SNP is a place in the DNA where one individual's genetic code may be slightly different from another's. Each individual has many SNPs, and together can contribute to disease susceptibility. In addition to SNPs observed on lcWGS, we examine millions of other SNPs that you have by comparing your data to thousands of other genomes, a statistical process known as imputation.

Step 2: CAD polygenic score development
Genome-Wide Association Studies or GWAS compared every common SNP in tens of thousands of people with and without CAD. This analysis produced for each SNP a statistical association with CAD, some SNPs increase risk and others decrease risk. We have developed and tested multiple scores in hundreds of thousands of people to find the best one.[7] About 6 million SNPs are included in this score. The CAD polygenic score in a single individual represents the sum of the risk related to each of the SNPs present in your DNA.

Step 3: Calculating your score
We calculate your score by adding the increased or decreased risks related to each of the SNPs present in your DNA. We transform your score into a percentile to determine how your score compares to others in the population with a similar racial/ethnic background. The distribution of scores in a population is a "bell-shaped" curve, with most people in the middle (average risk), and fewer people in the upper tail (higher risk) and lower tail (lower risk).

References and Citations

1. Genetic risk, adherence to a healthy lifestyle, and coronary disease. NEJM: 375, 2349-2358 (2016).
2. Polygenic risk score identifies subgroup with higher burden of atherosclerosis and greater relative benefit from statin therapy in the primary prevention setting. Circulation 135, 2091-2101 (2017).
3. Life's Simple 7. American Heart Association. https://www.heart.org/en/professional/workplace-health/lifes- simple-7
4. Ideal cardiovascular health and mortality from all causes and diseases of the circulatory system among adults in the United States. Circulation 125, 987-995 (2012).
5. Genetic Information Nondiscrimination Act of 2008. http://www.ginahelp.org
6. Low coverage whole genome sequencing enables accurate assessment of common variants and calculation of genome-wide polygenic scores. Genome medicine, 11(1), 1-12.
7. Genome-wide polygenic scores for common diseases identify individuals with risk equivalent to monogenic mutations. Nat Genetics, 50, 1219-1224 (2018).

This educational tool related to a polygenic score was developed at the Broad Institute of MIT and Harvard.
